# Surgical hand hygiene and febrile urinary tract infections in endourological surgery: a single-centre prospective cohort study

**DOI:** 10.1038/s41598-020-71556-z

**Published:** 2020-09-03

**Authors:** Rei Unno, Kazumi Taguchi, Yasuhiro Fujii, Naoko Unno, Shuzo Hamamoto, Ryosuke Ando, Akihiro Nakane, Atsushi Okada, Hiroyuki Kamiya, Takahiro Yasui

**Affiliations:** 1 Department of Nephro-urology, Nagoya City University Graduate School of Medical Sciences, 1 Kawasumi, Mizuho-cho, Mizuho-ku, Nagoya, Aichi 467-8601 Japan; 2 Department of Urology, Daido Hospital, Nagoya, Japan

**Keywords:** Infectious diseases, Urogenital diseases

## Abstract

Surgical hand hygiene reduces the risk of surgical site infections (SSIs). SSIs are not considered an issue in endourological surgery, whereas febrile urinary tract infections (f-UTIs) and urological sepsis are becoming problematic. We wondered whether surgical hand hygiene is necessary for endourological surgery. Therefore, we aimed to evaluate the influence of surgical hand hygiene on f-UTI onset in endourological surgery by comparing procedures in which surgical hand hygiene with double gloving was used with those in which regular hand hygiene with double gloving was used between April 2016 and July 2020. In this prospective cohort study of 477 patients who underwent endourological surgeries, surgeons in the surgical hand hygiene and regular hygiene groups performed surgery on 259 and 218 patients, respectively. There was no significant difference in patient background, and multivariate analyses revealed no significant differences in f-UTI onset (odds ratio, 0.87; *p* = 0.74) between the two groups. In conclusion, regular hand hygiene with double gloving may be considered an alternative to surgical hand hygiene to prevent endourological f-UTIs, which could alter operational protocols for endourological surgery. Further studies are needed to validate our findings.

## Introduction

Surgical hand hygiene, one of the most important factors affecting the risk of surgical site infections (SSIs), significantly reduces the bacterial count on the hands through mechanical removal of bacteria and involves the use of a sterilising solution. The traditional method of hand washing before surgery is a 3–5-min hand wash using a brush and an antimicrobial solution^1,2^. However, there is concern about skin damage from cleaning with a brush or disinfectant^3,4^; damaged skin can cause the further spread of infection. Recently, surgical hand disinfection with alcohol-based scrub solutions that do not require a brush or the like has become widespread^5^. In addition, there are several reports showing that these methods are more effective and more bactericidal than traditional hand scrubs^6,7^. Moreover, some reports have indicated that this method is preferred by staff engaged in surgery and that hand-rubbing is more effective^5,6,8,9^. Although an alcohol-based scrub has obvious advantages, many healthcare workers, including operative room nurses and surgeons, have been reported to have developed significant skin damage and allergies^10^.

In urological surgery in recent years, open surgery has decreased and endoscopic surgery has become mainstream. Compared to other open surgical procedures, endourological surgery does not directly involve touching the surgical site when using endoscopic devices, and appropriate perfusion during surgery has been shown to reduce the rate of infection (although an increase in perfusion pressure may increase the risk of infection). Therefore, SSIs are not considered an issue in endourological surgery, whereas febrile urinary tract infections (f-UTIs) and urological sepsis are becoming problematic. This situation raises the question whether hand sanitisation similar to that used in other surgical procedures is necessary. Urologists perform various endoscopic examinations and treatments such as placement of ureteral stents and nephrostomy, and some surgeons perform these procedures wearing single clean gloves or double gloves without involving hand hygiene. Although an earlier study described how careful hand washing and wearing sterile gloves are essential in urological endoscopic surgery^11^, there is no description of the exact method or incidence of infection.

Therefore, we aimed to evaluate the influence of surgical hand hygiene on f-UTI onset in endourological surgery through comparing procedures in which surgical hand hygiene had been used with those in which regular hand hygiene had been used. This is the first study to analyse and report whether omitting surgical hand hygiene may increase SSI in endourological surgery.

## Results

A total of 477 patients were enrolled during this study period, comprising 194 patients who underwent transurethral resection of a bladder tumour (TURBT), 189 who underwent ureteroscopy for a stone (URS), and 93 who underwent endoscopic combined intrarenal surgery (ECIRS). Surgeons in the surgical hand hygiene group and in the regular hygiene group performed surgery on 259 and 218 patients, respectively. There were no significant differences regarding patient history, such as the prevalence of organ infection, diabetes, history of smoking, steroid therapy, obesity, age, and malnutrition between the two groups (Table 1). Furthermore, no difference was found between the two groups in terms of patient history and operative and perioperative information details (Table 2). No differences were observed between the two groups in terms of postoperative fever, f-UTIs, inflammatory parameters of serum and urine, bacteriuria, duration of hospitalisation, and onset of sepsis.

**Table 1:** Patient background.

	Surgical hand hygiene group	Regular hand hygiene group	*p* value
	n = 259	n = 218	
Diabetes (%)	14.3	16.5	0.52
Smoking (%)	18.5	15.6	0.46
Steroid (%)	1.5	1.4	1.00
Obesity (%)	41.3	44.5	0.51
Age (years)	62.8 ± 17.1	62.0 ± 16.5	0.64
Malnutrition (serum albumin: mg/dL)	3.97 ± 0.49	4.07 ± 0.42	0.14

Obesity: BMI > 25 kg/m^2^.

Data (age and malnutrition) are expressed as mean ± SD. Student’s t-tests, Mann–Whitney-U tests, and chi-squared tests were used to determine statistical significance. A *p* value < 0.05 was considered statistically significant.

*BMI* body mass index, *SD* standard deviation.

**Table 2:** Operative and perioperative information.

	Surgical hand hygiene group	Regular hand hygiene group	*p* value
	n = 259	n = 218	
ASA.PS (%)			
1	117 (45.2)	91 (41.7)	0.30
2	125 (48.3)	118 (54.1)	
3	17 (6.6)	9 (4.1)	
Sex: male (%)	186 (71.8)	153 (70.2)	0.76
Preoperative Cre (mg/dL)	0.86 (0.28, 9.54)	0.87 (0.08, 8.41)	0.19
Preoperative CRP (mg/dL)	0.10 (0.00, 14.80)	0.10 (0.00, 9.44)	0.47
Preoperative WBC (× 10^3^/µL)	6.00 (2.90, 21.10)	6.10 (2.60, 13.40)	0.48
Preoperative bacteriuria (%)	58 (22.4)	41 (22.3)	0.64
Preoperative fever (%)	17 (6.7)	9 (4.1)	0.31
Preoperative pyuria (%)	111 (43.7)	89 (41.4)	0.64
Preoperative symptom (%)	110 (42.5)	86 (39.4)	0.51
Preoperative ureter stent (%)	24 (9.3)	15 (6.9)	0.40
Operation type (%)			
TURBT	108 (41.7)	87 (39.9)	0.82
URS	103 (39.8)	86 (39.4)	
ECIRS	48 (18.5)	45 (20.6)	
Operation time (min)	55.0 (3.0, 248.0)	47.0 (3.0, 216.0)	0.14
Total stone size (mm^2^)	50.6 (0.0, 34,213.9)	43.9 (0.0, 4,127.3)	0.91
Tumour size (mm)	8.8 (2.0, 50.0)	13.0 (2.0, 40.0)	0.23
Tumour number (%)			
1	61 (56.5)	53 (60.9)	0.83
2	15 (13.9)	11 (12.6)	
≧3	32 (29.6)	23 (26.4)	
Postoperative Cre (mg/dL)	0.85 (0.08, 7.57)	0.88 (0.37, 9.81)	0.19
Postoperative CRP (mg/dL)	0.60 (0.00, 16.87)	0.57 (0.00, 19.43)	0.33
Postoperative WBC (× 10^3^/µL)	7.90 (2.60, 30.30)	7.60 (1.50, 22.70)	0.31
Postoperative bacteriuria (%)	32 (12.3)	26 (11.9)	0.89
Postoperative fever (%)	21 (8.3)	19 (8.8)	0.87
Postoperative f-UTIs (%)	16 (6.2)	14 (6.4)	1.00
Postoperative sepsis (%)	5 (1.9)	7 (3.2)	0.39
Postoperative hospitalisation (days)	4.0 (1.0, 22.0)	3.0 (1.0, 16.0)	0.30
Postoperative ureter stent (%)	139 (53.9)	121 (55.5)	0.78
Postoperative nephrostomy (%)	18 (6.9)	17 (7.8)	0.72

Student’s t-test, Mann–Whitney-U test, and chi-squared test were performed. A *p* value < 0.05 was considered statistically significant.

*ASA PS* American Society of Anesthesiologists’ physical status, *Cre* creatinine, *CRP* C-reactive protein, *TURBT* transurethral resection of a bladder tumour, *WBC* white blood cell, f-UTIs: febrile urinary tract infections.

Multivariate analyses demonstrated that there was no significant association between surgical hand hygiene and the incidences of f-UTIs, sepsis, and inflammatory markers (serum CRP and blood WBC) (Tables 3 and 4). A preoperative rise in inflammatory markers, the presence of pyuria, having ureter stent, and long-term surgery were significantly associated with f-UTI-related factors. The length of hospitalisation significantly increased for elderly patients, for long-term surgery patients, and for patients with postoperative nephrostomy, whereas patients with a postoperative ureter stent had significantly shorter hospitalisation durations. Similarly, for patients who underwent urinary stone surgery, performing or not performing surgical hand hygiene showed no significant association with the onset of f-UTIs. In contrast, in patients who underwent URS with a preoperative ureter stent, the incidences of f-UTIs and sepsis increased significantly, whereas in patients who underwent URS with a postoperative ureter stent, the onset of f-UTIs, an inflammatory marker and the period of postoperative hospitalisation decreased significantly. No patients in either group developed an SSI by skin incision following ECIRS.

**Table 3:** Risk for f-UTIs development (postoperative fever, sepsis, and bacteriuria).

	Postoperative f-UTIs		Postoperative sepsis	
	Odds ratio (95% CI)	*p* value	Odds ratio (95% CI)	*p* value
**Total**				
Age	1.01 (0.98–1.04)	0.48	1.00 (0.95–1.05)	0.95
BMI	1.00 (0.92–1.08)	0.99	1.02 (0.94–1.10)	0.65
Surgical hand hygiene	0.87 (0.38–2.01)	0.74	0.74 (0.20–2.74)	0.66
Preoperative CRP	1.26 (1.03–1.56)	0.02	1.57 (1.21–2.04)	< 0.01
Preoperative WBC	1.11 (0.92–1.34)	0.29	1.02 (0.76–1.38)	0.88
Preoperative pyuria	3.83 (1.44–10.20)	< 0.01	8.53 (1.30–55.80)	0.02
Preoperative ureteral stent	3.73 (1.34–10.40)	0.01	3.14 (0.73–13.60)	0.13
Operation time	1.02 (1.01–1.03)	< 0.01	1.01 (0.99–1.02)	0.3
Postoperative ureteral stent	1.02 (0.34–3.05)	0.97	4.76 (0.43–53.50)	0.21
Postoperative nephrostomy	1.64 (0.49–5.46)	0.42	3.37 (0.58–19.70)	0.18
**URS**				
Surgical hand hygiene	1.48 (0.37–5.89)	0.57	1.65 (0.13–20.40)	0.7
Operation time	1.02 (1.00–1.05)	0.03	0.98 (0.91–1.05)	0.57
Total stone size	1.00 (0.99–1.00)	0.45	0.99 (0.94–1.03)	0.06
Preoperative ureteral stent	19.30 (4.04–92.00)	< 0.01	10.70 (1.16–133.00)	< 0.01
Postoperative ureteral stent	0.07 (0.01–0.89)	0.04	2.13 (0.00–4.12)	0.49
**ECIRS**				
Surgical hand hygiene	0.52 (0.15–1.75)	0.29	0.32 (0.06–1.73)	0.18
Operation time	1.00 (0.99–1.02)	0.68	1.00 (0.98–1.02)	0.82
Total stone size	1.00 (1.00–1.00)	0.52	1.00 (1.00–1.00)	0.80
Postoperative ureteral stent	1.19 (0.21–6.74)	0.84	1.72 (0.16–18.40)	0.65
Postoperative nephrostomy	1.79 (0.52–6.18)	0.36	2.43 (0.51–11.60)	0.26

With respect to postoperative f-UTIs and sepsis, multivariable analyses were performed for patient age, BMI, surgical hand hygiene, preoperative CRP, pyuria, WBC count, operation time, postoperative indwelling ureter stent, and nephrostomy using logistic regression analysis. Regarding URS and ECIRS, multivariable analyses were performed to compare above perioperative f-UTIs and sepsis in surgical hand hygiene, operation time, total stone size, and the preoperative and/or postoperative indwelling of ureter stent and nephrostomy. A *p* value < 0.05 was considered statistically significant.

*f-UTIs* febrile urinary tract infections, *BMI* body mass index, *CI* confidence interval, *CRP* C-reactive protein, *ECIRS* endoscopic combined intrarenal surgery, *f-UTIs* febrile urinary tract infections, *SE* standard error, *URS* ureteroscopy for a stone, *WBC* white blood cell.

**Table 4:** Risk for f-UTI development (postoperative increase in CRP, WBC, and hospitalisation).

	Postoperative CRP increase			Postoperative WBC increase			Postoperative hospitalisation		
	coefficient (95% CI)	SE	*p* value	Coefficient (95% CI)	SE	*p* value	Coefficient (95% CI)	SE	*p* value
**Total**									
Age	0.01 (− 0.01–0.02)	0.01	0.48	− 0.01 (− 0.03–0.01)	0.01	0.27	0.02 (0.01–0.03)	0.01	< 0.01
BMI	0.00 (− 0.02–0.02)	0.01	0.87	0.01 (− 0.02–0.04)	0.02	0.67	0.00 (− 0.02–0.02)	0.01	0.69
Surgical hand hygiene	− 0.13 (− 0.58–0.31)	0.22	0.55	0.32 (− 0.27–0.9)	0.3	0.29	0.18 (− 0.19–0.56)	0.19	0.33
Preoperative CRP	0.47 (0.29–0.66)	0.09	< 0.01	0.04 (− 0.2–0.28)	0.12	0.73	0.07 (− 0.1–0.23)	0.08	0.42
Preoperative WBC	0.09 (− 0.03–0.21)	0.06	0.15	0.53 (0.37–0.69)	0.08	< 0.01	− 0.07 (− 0.18–0.03)	0.05	0.17
Preoperative pyuria	0.12 (− 0.34–0.57)	0.23	0.61	0.02 (− 0.59–0.62)	0.31	0.95	− 0.01 (− 0.4–0.39)	0.2	0.97
Preoperative ureteral stent	1.13 (0.31–1.95)	0.42	< 0.01	0.04 (− 1.05–1.13)	0.55	0.95	0.42 (− 0.29 to − 1.13)	0.36	0.25
Operation time	0.01 (0–0.01)	0	0.02	0.02 (0.01–0.03)	0	< 0.01	0.02 (0.01–0.02)	0	< 0.01
Postoperative ureteral stent	0.53 (− 0.02–1.09)	0.28	0.06	− 0.34 (− 1.08–0.41)	0.38	0.37	− 0.49 (− 0.95–0.02)	0.24	0.04
Postoperative nephrostomy	0.60 (− 0.24–1.44)	0.43	0.16	− 0.54 (− 1.66–0.57)	0.57	0.34	1.67 (0.89–2.44)	0.4	< 0.01
**URS**									
Surgical hand hygiene	0.62 (− 0.33–1.57)	0.48	0.20	0.66 (− 0.83–2.15)	0.75	0.38	0.58 (− 0.12–1.28)	0.35	0.10
Operation time	0.00 (− 0.01–0.01)	0.01	0.97	0.01 (− 0.01–0.03)	0.01	0.29	0.01 (0–0.03)	0.01	0.04
Total stone size	0.00 (0–0.01)	0	0.42	0.00 (0–0.02)	0	0.15	0.00 (0–0.02)	0	0.39
Preoperative ureteral stent	1.95 (0.9–3)	0.53	< 0.01	− 0.77 (− 2.46–0.92)	0.85	0.37	1.03 (0.04–2.03)	0.5	0.04
Postoperative ureteral stent	− 2.10 (− 4.34–0.13)	1.13	0.06	− 5.65 (− 9.26 to − 2.04)	1.82	< 0.01	− 3.65 (− 5.62 to − 1.69)	1	< 0.01
**ECIRS**									
Surgical hand hygiene	− 0.46 (− 1.97–1.06)	0.76	0.55	0.08 (− 1.3–1.46)	0.69	0.91	− 0.15 (− 0.98–0.68)	0.42	0.73
Operation time	− 0.01 (− 0.03–0.01)	0.01	0.37	0.00 (− 0.01–0.02)	0.01	0.57	0.01 (0–0.02)	0.01	0.07
Total stone size	0.00 (0–0.01)	0	0.19	0.00 (0–0.01)	0	0.49	0.00 (0–0.01)	0	0.11
Postoperative ureteral stent	1.96 (− 0.23–4.15)	1.1	0.07	− 0.38 (− 2.38–1.61)	1	0.71	− 0.15 (− 1.35–1.04)	0.6	0.80
Postoperative nephrostomy	0.88 (− 0.82–2.58)	0.86	0.31	− 1.47 (− 3.02–0.08)	0.78	0.06	1.43 (0.5–2.37)	0.47	< 0.01

With respect to postoperative increase in CRP, WBC count, and the period of hospitalisation, multivariate analyses were performed for patient age, BMI, surgical hand hygiene, preoperative CRP, pyuria, WBC count, indwelling Foley catheter and ureter stent, operation time, postoperative indwelling ureter stent, and nephrostomy using liner regression analysis. Regarding URS and ECIRS, multivariate analyses were performed to compare the above perioperative parameters related to f-UTIs in surgical hand hygiene, operation time, total stone size, and the preoperative and/or postoperative indwelling of ureter stent and nephrostomy. A *p* value < 0.05 was considered statistically significant.

*BMI* body mass index, *CI* confidence interval, *CRP* C-reactive protein, *ECIRS* endoscopic combined intrarenal surgery, *f-UTIs* febrile urinary tract infections, *SE* standard error, *URS* ureteroscopy for a stone, *WBC* white blood cell.

## Discussion

Our study findings showed that omitting surgical hand hygiene before endourological surgery had no significant association with an increase in the onset of f-UTIs and the period of hospitalisation. The onset of f-UTIs and a longer hospitalisation period post-endourological surgery were mainly dependent on the presence of preoperative inflammatory markers and pyuria; these results were the same even when limited to surgery for urinary stones.

Disinfection may be the single most important development that contributes to successful surgical outcomes in modern times. Disinfection technology is a standard approach that all surgeons and surgical assistants are familiar with; Silvia et al. indicated that health care workers cleaned their hands, on an average, 5–42 times per shift and 1.7–15.2 times per hour^12^. Joseph Lister recognised that surgical outcomes could be improved through hand antisepsis^13^. Over the ensuing decades, additional reductions in infection came about with the development of sterile sutures, gloves, gowns, drapes, and surgical hats, increased cleanliness of operating rooms, and improvement in sterilisation techniques for surgical instruments^14–16^. Even with such progress, hand washing, which has been a key component of this technology for more than 100 years, remains integral to preventing infection at the surgical site and is cost effective.

Many comparative studies using various types of disinfectant and waterless hand rubs instead of disinfectants have been undertaken to evaluate SSI prevention. Some reports showed the superiority of waterless hand rub products over traditional hand-scrubbing products^17,18^. Ho et al. showed that waterless hand rubs showed stronger microbiocidal effects than the povidone iodine (PI) scrub, although there was no significant difference between the waterless hand rubs and the chlorhexidine scrub regard to antibacterial effect^19^. Weight et al. reported that Avagard hand antisepsis was more effective than a traditional scrub in paediatric urological surgery^20^.

However, in endourological surgery where SSI is not considered an issue but f-UTIs are a surgery-related infection issue, few reports have considered the need for preoperative surgical hand hygiene^11^. Furthermore, many reports have shown that an extended operative time may increase the risk of infection^21^, which means that the incidence of infection is likely to be reduced during surgery of short duration. Given that the time required for endourological surgery tends to be short, this raises the question whether preoperative surgical hand hygiene needs to be performed, as in other surgical procedures. Recently, double gloving has been recommended to prevent occupational infection^22^. In our study, the use of double gloves after regular hand hygiene did not result in the onset of f-UTIs or an increase in the hospitalisation period. As in previous reports, our results demonstrated that the preoperative rise in inflammatory markers and the presence of pyuria were significantly associated with f-UTI-related factors and longer hospitalisations.

Although not considered in our study, some reports have shown the time efficacy and cost-effectiveness of not applying surgical hand hygiene. Most traditional scrub methods require at least 3–5 min. If this procedure is omitted, surgical hand preparation takes less time and it reduces the time required for performing hand hygiene practices^23^. Weight et al. indicated that excluding the cost of dry towels and water after scrubbing, using Avagard for hand disinfection is half the cost of disinfecting using a hand brush^20^. Furthermore, brushless disinfection does not generate waste such as brushes and dry towels. Another report found that the cost of a waterless hand scrub is one-third that of traditional hand washing (USD 20 vs. USD 60)^3^. Considering these reports, it seems that regular hand hygiene can be performed in a shorter time and at a lower cost.

There are several limitations to our study. First, although the results of the perioperative analysis divided by each surgeon showed almost no significant difference between the surgeons (Supplemental Table S1), this single-centre, non-randomised study might have a selection bias owing to facility specific cases and surgeon’s preference of the type of hand hygiene methods. Second, we could not measure the colony-forming unit count with respect to the hands and endoscopic apparatus, which enables a more accurate assessment of hand hygiene and may have revealed any potential micro-punctures of the gloves associated with SSI onset. Third, ECIRS differed from other procedures in that this procedure required at least two working surgeons and a small skin incision. Fourth, in this study, we utilised axillary temperature, which is 0.5–1.0 °C lower than core temperature, as standard practice in our institutional protocol. Finally, the meaning difference proportion set when calculating the sample size might have been large, in which case, the examination would be underpowered.

We found that general hand hygiene with double gloving may be considered as an alternative to surgical hand hygiene to prevent endourological f-UTIs. These results, involving real-world data reflecting our daily practice, need further validation but could change the operational protocol for endourological surgery, especially surgery of relatively short duration, with an improvement to the operating room environment. While patient numbers were not sufficiently large to reach a definitive conclusion, this is the first report indicating that using general hand hygiene as an alternative to surgical hand hygiene may save time and be cost-effective for endourological surgical staff.

## Methods

### Study design

This was a single-centre prospective cohort study, comparing f-UTIs onset involving endourological surgeries undertaken from April 2016 to July 2020. Patients who underwent TURBT, URS, and ECIRS were enrolled. To avoid potential bias, each procedure was performed by 4 certified attending surgeons. We analysed data concerning patient history, using the American Society of Anesthesiologists—physical status (ASA PS) scores in relation to the following: sex, body mass index (BMI), parameters related to SSI (infection site: surgical site or non-surgical site). Data on diabetes, smoking, steroid use, obesity (BMI > 25), age, malnutrition (serum albumin levels), and operation details (operation time, total stone size, tumour size, and tumour number) were also analysed. Perioperative information, such as preoperative serum creatinine (Cre) levels, serum C-reactive protein (CRP) levels, white blood cell (WBC) count, bacteriuria, fever (axillary temperature > 37 °C), pyuria, symptoms, and ureter stent, were obtained. Postoperative data such as postoperative bacteriuria, fever (axillary temperature > 38 °C), sepsis, hospitalisation, serum Cre levels, serum CRP levels, WBC count, indwelling of ureter stent, and nephrostomy were also obtained. Sepsis was diagnosed according to international consensus definitions^24^. To evaluate a complication following endourological surgery, f-UTI was set as a primary endpoint and postoperative hospitalisation was set as a secondary endpoint. We investigated the association between surgical hand hygiene or regular hand hygiene and postoperative f-UTIs, sepsis, and increase of serum CRP and WBC count as perioperative parameters related to f-UTI onset. Patients who had a long-term indwelling catheter were excluded from the analysis. All procedures used in this study were approved by the Ethical Committee of Daido Hospital (ECD2019019), and informed consent for treatment was obtained from all patients. All methods/experiments were carried out in accordance with relevant guidelines and regulations (Declaration of Helsinki).

### Clinical paths

In the pre- and peri-operative period of TURBT, URS, and ECIRS, clinical paths (Supplemental Table S2) were used to examine and treat according to the protocol, regardless of the operator.

### Surgical methods

In advance, every certified attending surgeon made a decision whether or not surgical or regular hand hygiene was to be undertaken, and two groups were formed accordingly. In the surgical hand hygiene group, either hand-scrubbing or hand-rubbing methods were used in addition to wearing double gloves (donning one pair of gloves then, after gowning, donning a second pair of gloves). The surgeons undertaking hand-scrubbing were required to adhere to the following protocol: (1) run warm water and wash hands and arms with medicated soap (e.g., chlorhexidine- or povidone iodine-containing soaps) using hand brushes; (2) rinse both hands and arms with running warm water, and; (3) wipe hands and arms with sterile towels and/or sterile paper. The hand-rubbing (waterless) method was as follows: (1) wash hands using non-medicated soap and warm water, (2) wipe hands and arms with non-sterilised towels/paper (3) use alcohol-based hand rubs for both the hands and the arms, and (4) wait until the hands dry. The surgeons in the regular hygiene group were required to adhere to the following protocol: (1) wash hands using non-medicated soap and warm water; (2) wipe hands and arms with non-sterilised towels/paper, and; (3) wear double gloves. In each method, we used double gloves to prevent contamination owing to micro-rupture.

### Calculation of sample size

Previous studies have reported that the rate of f-UTIs in endourological surgery was < 5%^25^, but we included ECIRS cases in this study. In ECIRS cases, f-UTIs onset is reported up to 10–20%^26,27^; based on that and considering that the ratio of ECIRS in endourological surgery at our facility was approximately 10%, we set the incidence of f-UTIs to 5% in the surgical hand hygiene group, and to 7.5% in the regular hand hygiene group because of increased risk of infection owing to omission of conventional disinfection practices. The difference in the incidence of f-UTI due to the presence or absence of surgical hand washing was within a permissible range of up to 4%, the detection power was 80%, the significance level was 5% on two side, and we calculated the sample size for comparison of the ratio of two groups (non-inferiority) according to a statistician's consultation. Consequently, the minimum required number of samples for each group was 218, and the appropriate number of participants were determined accordingly.

### Statistical methods

All data are expressed as mean ± standard deviation (SD). Student’s t-test, Mann–Whitney-U test, and chi-square test were performed. Concerning postoperative fever, sepsis, bacteriuria, increases in CRP and WBC count, and the period of hospitalisation, multivariate analyses were performed in relation to patient age, BMI, surgical hand hygiene, preoperative CRP, WBC count, pyuria, the use of an indwelling Foley catheter and ureter stent, operation time, and postoperative indwelling of ureter stent and nephrostomy, using logistic regression analysis and linear regression analysis. Regarding URS and ECIRS, multivariate analyses were performed to compare the above perioperative parameters related to f-UTIs and the period of hospitalisation, in relation to surgical hand hygiene, operation time, total stone size, and the preoperative and/or postoperative indwelling ureter stent and nephrostomy. A *p* value < 0.05 was considered statistically significant. All statistical analyses were performed with EZR (Saitama Medical Center, Jichi Medical University, Saitama, Japan), which is a graphical user interface for R (The R Foundation for Statistical Computing, Vienna, Austria)^28^.

## Supplementary information

Supplementary information
