# Supplementary material for: Surgical hand hygiene and febrile urinary tract infections in endourological surgery: a single-centre prospective cohort study
Source: Sci Rep. 2020 Sep 3;10:14520. doi: 10.1038/s41598-020-71556-z (PMC7471674; doi:10.1038/s41598-020-71556-z)
Supplement: Supplementary file 1 — Supplementary information [file 41598_2020_71556_MOESM1_ESM.docx]

**Surgical hand hygiene and febrile urinary tract infections in endourological surgery: A single-centre prospective cohort study**

Rei Unno^a,b^, Kazumi Taguchi^a,b*^, Yasuhiro Fujii^b^, Naoko Unno^a^, Shuzo Hamamoto^a^, Ryosuke Ando^a^, Akihiro Nakane^a^, Atsushi Okada^a^, Hiroyuki Kamiya^b^, Takahiro Yasui^a^

^a^Department of Nephro-urology, Nagoya City University Graduate School of Medical Sciences, Aichi, Japan

^b^Department of Urology, Daido Hospital

*Corresponding author

Department of Nephro-urology, Nagoya City University Graduate School of Medical Sciences, 1 Kawasumi, Mizuho-cho, Mizuho-ku, Nagoya 467-8601, Japan

Tel: +81-52-853-8266; Fax: +81-52-852-3179

Email: ktaguchi@med.nagoya-cu.ac.jp

**Supplemental Table S1. Operative and perioperative information examined by each surgeon**

|  | Surgical hand hygiene group | | Regular hand hygiene group | |  |
| --- | --- | --- | --- | --- | --- |
| operator | Y.F. | H.K. | R.U. | K.T | p value |
| n | 124 | 135 | 105 | 113 |  |
| ASA.PS (%) |  |  |  |  | 0.28 |
| 1 | 60 ( 48.4) | 57 ( 42.2) | 41 ( 39.0) | 50 ( 44.2) |  |
| 2 | 53 ( 42.7) | 72 ( 53.7) | 59 ( 56.2) | 59 ( 52.2) |  |
| 3 | 11 ( 8.9) | 6 ( 4.5) | 5 ( 4.8) | 4 ( 3.5) |  |
| sex: male (%) | 87 ( 70.2) | 99 ( 73.3) | 74 ( 70.5) | 79 ( 69.9) | 0.92 |
| preoperative Cre (mg/dL) | 0.87 [0.47, 9.54] | 0.85 [0.28, 9.46] | 0.87 [0.10, 8.41] | 0.87 [0.08, 1.71] | 0.79 |
| preoperative CRP (mg/dL) | 0.10 [0.00, 6.60] | 0.10 [0.00, 14.80] | 0.10 [0.00, 9.44] | 0.08 [0.01, 5.72] | 0.46 |
| preoperative WBC (x10^3^/µL) | 5.85 [3.40, 12.90] | 6.00 [2.90, 21.10] | 6.20 [2.60, 13.40] | 6.00 [3.30, 11.00] | 0.79 |
| preoperative bacteriuria (%) | 28 ( 23.7) | 30 ( 25.0) | 21 ( 24.1) | 20 ( 20.6) | 0.89 |
| preoperative fever (%) | 10 ( 8.1) | 7 ( 5.4) | 6 ( 5.7) | 3 ( 2.7) | 0.35 |
| preoperative pyuria (%) | 50 ( 41.0) | 61 ( 46.2) | 47 ( 45.2) | 42 ( 37.8) | 0.54 |
| preoperative symptom (%) | 64 ( 51.6) | 46 ( 34.1) | 44 ( 41.9) | 42 ( 37.2) | 0.02 |
| preoperative ureter stent (%) | 10 ( 8.1) | 14 ( 10.4) | 6 ( 5.7) | 9 ( 8.0) | 0.63 |
|  |  |  |  |  |  |
| operation type (%) |  |  |  |  | 0.99 |
| TURBT | 53 ( 42.7) | 55 ( 40.7) | 43 ( 41.0) | 44 ( 38.9) |  |
| URS | 49 ( 39.5) | 54 ( 40.0) | 40 ( 38.1) | 46 ( 40.7) |  |
| ECIRS | 22 ( 17.7) | 26 ( 19.3) | 22 ( 21.0) | 23 ( 20.4) |  |
| operation time (min) | 60.0 [3.0, 248.0] | 50.0 [9.0, 191.0] | 44.0 [11.0, 213.0] | 49.0 [3.0, 216.0] | 0.33 |
| total stone size (mm^2^) | 50.6 [0.0, 11397.9] | 50.2 [0.0, 34213.9] | 42.3 [0.0, 4127.3] | 45.5 [0.0, 3695.3] | 0.99 |
| tumour size (mm) | 7.9 [3.0, 35.0] | 10.0 [2.0, 50.0] | 15.0 [2.0, 40.0] | 10.5 [2.0, 30.0] | 0.70 |
| tumour number (%) |  |  |  |  | 0.66 |
| 1 | 26 (49.1) | 35 (63.6) | 25 (58.1) | 28 (63.6) |  |
| 2 | 7 (13.2) | 8 (14.5) | 6 (14.0) | 5 (11.4) |  |
| ≧3 | 20 (37.7) | 12 (21.8) | 12 (27.9) | 11 (25.0) |  |
|  |  |  |  |  |  |
| postoperative Cre (mg/dL) | 0.87 [0.46, 7.57] | 0.83 [0.08, 6.17] | 0.88 [0.37, 7.31] | 0.90 [0.45, 9.81] | 0.37 |
| postoperative CRP (mg/dL) | 0.63 [0.01, 10.13] | 0.60 [0.00, 16.87] | 0.59 [0.00, 9.32] | 0.47 [0.01, 19.43] | 0.81 |
| postoperative WBC (x10^3^/µL) | 7.70 [3.60, 30.30] | 8.30 [2.60, 21.90] | 7.60 [1.50, 14.80] | 7.55 [2.50, 22.70] | 0.29 |
| postoperative bacteriuria (%) | 12 (12.0) | 20 (17.1) | 13 (14.8) | 13 (13.4) | 0.74 |
| postoperative fever (%) | 10 ( 8.1) | 11 ( 8.6) | 9 ( 8.6) | 10 ( 8.9) | 0.99 |
| postoperative fUTIs (%) | 7 ( 5.7) | 9 ( 6.7) | 6 ( 5.7) | 8 ( 7.1) | 0.96 |
| postoperative sepsis (%) | 1 ( 0.8) | 4 ( 3.0) | 3 ( 2.9) | 4 ( 3.6) | 0.54 |
| hospitalization (days) | 4.0 [1.0, 22.0] | 3.0 [1.0, 13.0] | 3.0 [1.0, 12.0] | 3.0 [1.0, 16.0] | 0.40 |
| postoperative ureter stent (%) | 67 ( 54.0) | 72 ( 53.7) | 56 ( 53.3) | 65 ( 57.5) | 0.91 |
| postoperative nephrostomy (%) | 11 ( 8.9) | 7 ( 5.2) | 10 ( 9.5) | 7 ( 6.2) | 0.51 |

Student’s t-test, Mann-Whitney-U test, and chi-squared test were performed. A p-value <0.05 was considered statistically significant.

Abbreviations: ASA PS: American Society of Anesthesiologists’ physical status, Cre: creatinine, CRP: C-reactive protein, TURBT: transurethral resection of a bladder tumor, WBC: white blood cell, f-UTIs: febrile urinary tract infections

**Supplemental Table S2. Clinical Path**

1. **TURBT**

|  |  | preoperative period | |  | on the day of surgery | |  | postoperative period | | |  | clinical visit |
| --- | --- | --- | --- | --- | --- | --- | --- | --- | --- | --- | --- | --- |
|  |  | 〜1 month before surgery | 1 day before surgery |  | before surgery | after surgery |  | 1st day after surgery | 2nd day after surgery | 3rd-4th day after surgery |  | 2-4 weeks after surgery |
| observation of condition |  | ○ | ○ |  | ○ | ○ |  | ○ | ○ | ○ |  | ○ |
| vital sign check |  | ○ | ○ |  | ○ | ○ |  | ○ | ○ | ○ |  | ○ |
| blood test |  | ○ |  |  |  |  |  | ○ |  |  |  |  |
| urinalysis |  | ○ |  |  |  |  |  | ○ |  |  |  | ○ |
| urine culture test |  | ○ |  |  |  |  |  |  |  |  |  |  |
| antibiotic administration |  |  |  |  | ○ | ○ |  | ○ |  |  |  |  |
|  |  |  |  |  |  |  |  |  | removal of a foley catheter | |  |  |
| hospitalisation |  |  |  |  |  |  |  |  |  |  |  |  |

1. **URS**

|  |  | preoperative period | |  | on the day of surgery | |  | postoperative period | |  | clinical visit |
| --- | --- | --- | --- | --- | --- | --- | --- | --- | --- | --- | --- |
|  |  | 〜1 month before surgery | 1 day before surgery |  | before surgery | after surgery |  | 1st day after surgery | 2nd -3rd day after surgery |  | 2-4 weeks after surgery |
| observation of condition |  | ○ | ○ |  | ○ | ○ |  | ○ | ○ |  | ○ |
| vital sign check |  | ○ | ○ |  | ○ | ○ |  | ○ | ○ |  | ○ |
| blood test |  | ○ |  |  |  |  |  | ○ |  |  |  |
| urinalysis |  | ○ |  |  |  |  |  | ○ |  |  | ○ |
| urine culture test |  | ○ |  |  |  |  |  |  |  |  |  |
| antibiotic administration |  |  |  |  | ○ | ○ |  | ○ |  |  |  |
|  |  |  |  |  |  |  |  | removal of a foley catheter  and a ureteral stent | |  |  |
| hospitalisation |  |  |  |  |  |  |  |  |  |  |  |

1. **ECIRS**

|  |  | preoperative period | |  | on the day of surgery | |  | postoperative period | | |  | clinical visit |
| --- | --- | --- | --- | --- | --- | --- | --- | --- | --- | --- | --- | --- |
|  |  | 〜1 month before surgery | 1 day before surgery |  | before surgery | after surgery |  | 1st day after surgery | 2nd day after surgery | 3rd-4th day after surgery |  | 2-4 weeks after surgery |
| observation of condition |  | ○ | ○ |  | ○ | ○ |  | ○ | ○ | ○ |  | ○ |
| vital sign check |  | ○ | ○ |  | ○ | ○ |  | ○ | ○ | ○ |  | ○ |
| blood test |  | ○ |  |  |  |  |  | ○ |  |  |  |  |
| urinalysis |  | ○ |  |  |  |  |  | ○ |  |  |  | ○ |
| urine culture test |  | ○ |  |  |  |  |  |  |  |  |  |  |
| antibiotic administration |  |  |  |  | ○ | ○ |  | ○ |  |  |  |  |
|  |  |  |  |  |  |  |  | removal of a foley catheter and a ureteral stent | |  |  |  |
|  |  |  |  |  |  |  |  | removal of a foley catheter and clamp of a nephrostomy tube | removal of a  nephrostomy tube | |  |  |
| hospitalisation |  |  |  |  |  |  |  |  |  |  |  |  |

In pre- and perioperative period of TURBT, URS, and ECIRS, we used clinical paths (as follows) to examined and treat according to the protocol regardless of the operator.

1. We observed the condition, checked vital sign, and performed blood test, urinalysis, and urine culture tests as preoperative test in the outpatient department within 1 month before the surgeries.
2. The patients were hospitalised the day before the surgeries. Then we observed the condition, checked the vital sign.
3. Just before surgery, all patients received antibiotics once before surgery (cefazolin was used when there was no preoperative pyuria, and antibiotics were selected according to the urine culture when pyuria was present).
4. After procedures, we indwelled a Foley catheter in all cases, and we placed a ureteral stent in URS, and placed either a ureteral stent or a nephrostomy tube in ECIRS.
5. After all surgery, the patients were hospitalised until at least the next day and antibiotics were administered about 6 hours after surgery.
6. Approximately 12 hours after the previous day's antibiotic administration, another antibiotic administration was given.
7. TURBT: A Foley catheter was removed on the second day after surgery and patients were discharged from the 3rd-4th postoperative day. URS: A Foley catheter and a ureteral stent were removed on the 1st postoperative day, and the patients were discharged from the 2nd-3rd postoperative day (depending on the residual stones, and condition of ureteral damage during surgery, some patients were discharged with a ureteral stent). ECIRS: A Foley catheter was removed, and ureteral stent was removed (depending on the condition of ureteral damage during surgery, some patients were discharged with ureteral stent) or a nephrostomy tube was clamped on the 1st postoperative day. A nephrostomy tube was removed on the second day, and the patients were discharged from the 3rd-4th postoperative day.
8. Regarding the timing of confirming the existence of f-UTIs, the day after surgery, we performed blood test, urinalysis, and check the vital sign. During the hospitalisation period, the vital signs were measured, and if patient had a fever, blood sampling, and urinalysis were examined as necessary. Furthermore, we confirmed the presence or absence of fever and other symptoms in the outpatient clinic about 2-4 weeks after surgery.
